# Observation of in-plane shear stress fields in off-axis SiC wafers by birefringence imaging

**DOI:** 10.1107/S1600576722006483

**Published:** 2022-07-30

**Authors:** Shunta Harada, Kenta Murayama

**Affiliations:** a Institute of Materials and Systems for Sustainability, Nagoya University, Furo-cho, Nagoya, 464-8601, Japan; b Mipox Corporation, 6-11-3, Nishishinjuku, Shinjuku-ku, Tokyo 160-0023, Japan; Australian Synchrotron, ANSTO, Australia

**Keywords:** birefringence, defect characterization, silicon carbide

## Abstract

For the nondestructive characterization of SiC wafers for power device application, birefringence imaging is one of the promising methods. In the present study, it is demonstrated that birefringence image contrast variation in off-axis SiC wafers corresponds to the in-plane shear stress under conditions slightly deviating from crossed Nicols according to both theoretical consideration and experimental observation. The current results indicate that the characterization of defects in SiC wafers is possible to achieve by birefringence imaging.

## Abstract 2

Theoretical consideration and experimental demonstration reveal that the contrast variation in a birefringence image of an off-axis SiC wafer corresponds to the in-plane shear stress field.

## 1. Introduction

Silicon carbide (SiC) is a promising material for power devices due to its superior physical properties, such as wide bandgap, high breakdown field strength, high saturation electron drift velocity and high thermal conductivity (Cooper *et al.*, 2002 ▸; Matsunami, 2006 ▸; Kimoto & Watanabe, 2020 ▸). Unlike silicon (Si), which can be produced dislocation free (Dash, 1959 ▸), commercial SiC wafers contain a lot of dislocations which adversely affect the performance, the reliability and the yield of the power devices (Neudeck & Powell, 1994 ▸; Kimoto, 2015 ▸). Therefore, it is important to characterize dislocations in SiC wafers. Among various kinds of characterization methods, including X-ray topography, photoluminescence imaging and molten potassium hydroxide (KOH) etching (Dudley *et al.*, 2003 ▸; Tajima *et al.*, 2006 ▸; Katsuno *et al.*, 1999 ▸), birefringence imaging using polarized light is a promising method for nondestructive characterization of SiC wafers (Ma *et al.*, 2002 ▸; Ouisse *et al.*, 2010 ▸; McGuire *et al.*, 2018 ▸; Kawata *et al.*, 2021 ▸). Birefringence image contrast of dislocations in various kinds of crystals has been investigated since the first observation of the stress field of dislocations in Si by Bond and Andrus (Bond & Andrus, 1956 ▸; Bullough, 1958 ▸; Maiwa *et al.*, 1989 ▸; Ming & Ge, 1990 ▸; Hoa *et al.*, 2014 ▸; Tanaka *et al.*, 2019 ▸). Observation of dislocations in crystalline materials by stress-induced birefringence was usually conducted along the principal optical axis with crossed Nicols, in which the polarization direction of the polarizer and analyzer is vertical. However, SiC wafers for power device fabrication are usually 4° off-oriented from the (0001) plane toward the [11 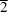 0] direction for step-controlled epitaxial growth, which is necessary for the suppression of polytype transformation during homoepitaxial SiC growth by chemical vapor deposition (CVD) (Ueda *et al.*, 1990 ▸; Matsunami & Kimoto, 1997 ▸; Kimoto *et al.*, 1993 ▸). In the present study, based on both theoretical consideration and experimental observation, we have demonstrated that birefringence image contrast variation in off-axis SiC wafers corresponds to the in-plane shear stress when the polarization direction of the analyzer is slightly deviated from crossed Nicols.

## 2. Theoretical modeling and calculation

In any given coordinate system, the equation of the index ellipsoid in an unstressed perfect hexagonal crystal is described as follows (Ge *et al.*, 1991 ▸):

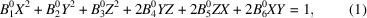

where 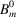 (*m* = 1, 2,…, 6) are the elements of the relative dielectric impermeability tensor, which is equal to the inverse of the dielectric tensor. Here we consider that the *Z* axis is tilted at θ from [0001] toward [11 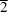 0], the *X* axis is [1 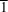 00], and the *Y* axis is perpendicular to the *X* and the *Z* axes in a hexagonal crystal, which corresponds to the observation of an off-angled SiC wafer. In this coordinate system, the off-diagonal element of *X* and *Y* vanishes ( 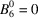 ) and the diagonal elements are as follows (Lin *et al.*, 2011 ▸):

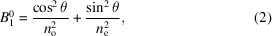




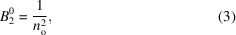




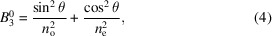

where *n*_o_ and *n*_e_ are reflective indices for ordinary and extraordinary light. When stress is applied to the crystal, the equation of the index ellipsoid is modified:

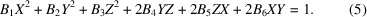

The small change of refractive index is produced by the local stress field, which is described by using piezooptical coefficients (π_*mn*_) and the local stress field (σ_*n*_) as follows:

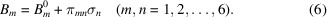

For light propagating along the *Z* axis, the secular equation that determines major and minor axes of the intersection ellipse is described as follows (Fathers & Tanner, 1973 ▸):

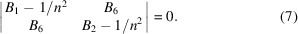

**Figure 1:**
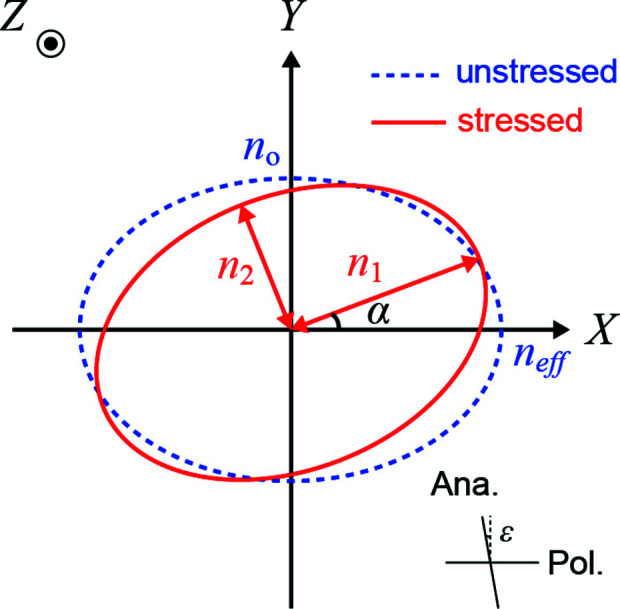
Schematic illustration of the intersection ellipse of the plane normal to the direction of light propagation for the unstressed and stressed crystal. Ana. and Pol. denote the orientations of the analyzer and polarizer.

From equation (7), two refractive indices (*n*_1_ and *n*_2_) are obtained, and the difference between *n*_1_ and *n*_2_ and the angle α between the *X* axis and the major axis of the ellipse is approximately given by (Ge *et al.*, 1991 ▸)

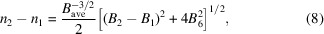




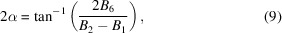

where *B*_ave_ is the average value of *B*_1_ and *B*_2_. Fig. 1 ▸ schematically illustrates the intersection ellipse of the plane normal to the direction of light propagation, the *Z* axis, with the index ellipsoid for an unstressed crystal [equation (1)] and a stressed crystal [equation (5)].

Here we consider observation under conditions slightly deviating from crossed Nicols, with the polarizer direction parallel to the *X* axis (Fig. 1 ▸). In this case the distribution of light intensity is as follows (Born & Wolf, 1980 ▸):

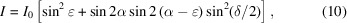

where *I*_0_ is the intensity of the incident polarized light and ɛ is the deviation from the crossed-Nicols condition, as shown in Fig. 1 ▸. δ is the phase retardation between two light beams with different refractive indices propagating in a specimen with a thickness of Δ*l* as follows:

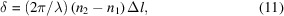

where λ is the wavelength of the incident light. Generally, the retardation of stress-induced birefringence is very small and here we assume sin(δ/2) = δ/2. In this case, the intensity distribution is expressed as follows:

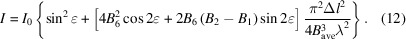




Here we note that (*B*_2_ − *B*_1_) is nearly equal to 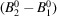 and much larger than *B*_6_ since the components of π_*mn*_σ_*n*_ are small for a long-range stress field of dislocations in an off-axis SiC wafer. Using the reported values (Wang *et al.*, 2013 ▸; Herms *et al.*, 2021 ▸), the estimated value of 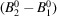 for 4H-SiC with θ = 4° corresponds to the value of *B*_6_ with σ_6_ = 84 MPa, which is much larger than the stress field of threading edge dislocations (TEDs) in 4H-SiC with an observation range of (<−1 MPa). Then, equation (12) is approximated as follows:

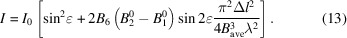

The first term, which represents background contrast in the birefringence image, is not dependent on the stress field and is always positive. On the other hand, the second term, which represents the contrast variation due to the local stress field, is proportional to *B*_6_ and σ_6_. Thus, when the midtone contrast level of the birefringence image is adjusted to the background contrast level, the contrast variation corresponds to an in-plane shear stress field distribution. From equation (13), a positive value of σ_6_ leads to a dark contrast and a negative value leads to a bright contrast. This is quite different from the formula of the birefringence contrast of dislocations viewed on an optically isotropic plane, in which the birefringence intensity is proportional to the difference between in-plane normal stress fields (Ge *et al.*, 1991 ▸; Pinto & Jones, 2009 ▸).

**Figure 2:**
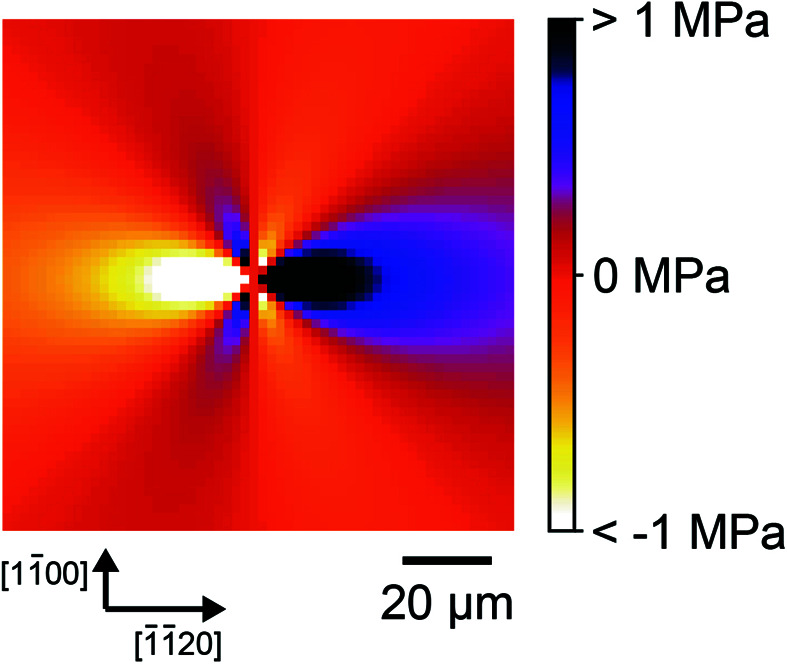
Calculated in-plane shear stress distribution of a TED with a Burgers vector of 1/3[ 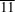 20].

To confirm the relationship between the birefringence image contrast and local stress field in off-axis SiC crystals, we have calculated the local stress field induced by a TED and compared the experimental birefringence image of the TED. For the calculation, we neglected the inclination of the TED and assumed that the incident direction is [0001] for simplicity. In this case, the in-plane shear stress field around the TED is expressed as follows (Chou, 1962 ▸):

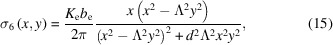

where b_e_ is the magnitude of the Burgers vector of the TED,

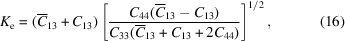




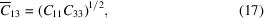




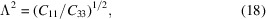




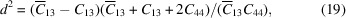

and *C*_*ij*_ denote the elastic constants in the standard coordinate system for a hexagonal lattice. For the calculation of the stress field, the elastic constants of 4H-SiC reported by Kamitani *et al.* (1998 ▸) were used. Fig. 2 ▸ shows the calculated in-plane shear stress field around a TED with a Burgers vector of 1/3[ 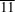 20]. Here we show the distribution of in-plane shear stress (σ_6_) with a color scale in which the positive values are dark and the negative values are bright for comparison with birefringence image contrast.

## 3. Materials and methods

A thick 4H-SiC epitaxial layer was grown by CVD on a 4H-SiC wafer with 4° off-cut toward [11 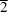 0]. Both sides of the crystal were planarized by chemical–mechanical polishing and the thickness of the crystal was about 150 µm. The birefringence imaging was conducted using an XS-1 microscope (Mipox Co. Ltd), which is a polarized light microscope with parallel irradiation. The wavelength of the incident light was 405 nm. The images were digitally recorded with a pixel resolution of about 1.8 µm per pixel. For the comparison, synchrotron X-ray topography images were taken at the same position as the birefringence image. Grazing-incidence reflection synchrotron topography using a monochromatic X-ray beam of 8.27 keV was conducted at BL8S2 of Aichi Synchrotron Radiation Center, Japan. The applied **g** vector was [11 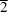 8]. The topography images were recorded on nuclear emulsion plates (Ilford) and digitally recorded by the optical microscope with transmission illumination.

## 4. Experimental confirmation

**Figure 3:**
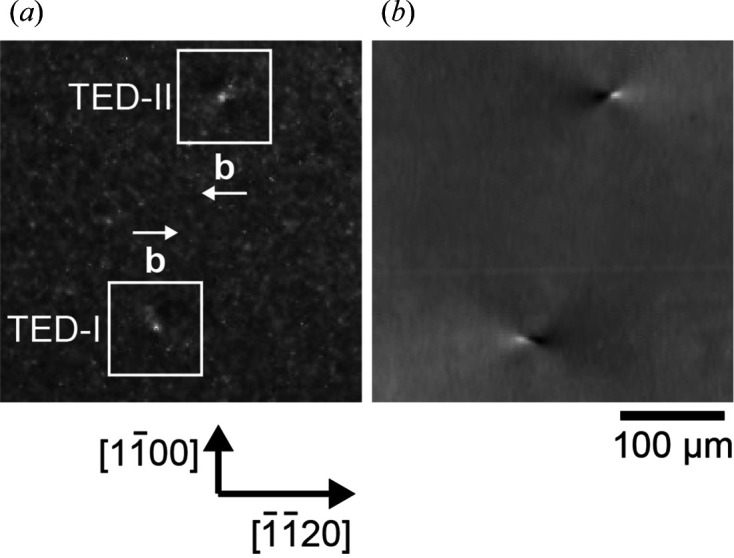
(*a*) X-ray topography image and (*b*) birefringence image under conditions slightly deviating from crossed Nicols (ɛ = 5°) taken at the same position on an SiC wafer (Δ*l* = 150 µm). The direction of the Burgers vector for TED-I is [ 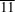 20] and that for TED-II is [11 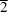 0].

Fig. 3 ▸ shows an X-ray topography image and birefringence image under conditions slightly deviating from crossed Nicols taken at the same position. In the X-ray topography image [Fig. 3 ▸(*a*)], small contrasts of TEDs were observed, and one can identify their Burgers vectors as 1/3[ 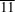 20] for TED-I and 1/3[11 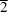 0] for TED-II judging from the characteristic contrast of TEDs depending on the direction of the Burgers vector (Kamata *et al.*, 2009 ▸; Harada *et al.*, 2014 ▸). At the same positions as the TED contrasts in the X-ray topography image, contrasts composed of dark and bright components were observed in the birefringence image [Fig. 3 ▸(*b*)]. The birefringence contrast of TED-I with the Burgers vector of 1/3[ 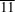 20] is similar to the calculated in-plane stress field shown in Fig. 2 ▸. Furthermore, the birefringence contrast of TED-II with the Burgers vector of 1/3[11 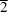 0], which is the opposite direction to the Burgers vector for TED-I, corresponds to an inversion of the stress field from Fig. 2 ▸. We also confirmed that the birefringence contrasts of other TEDs having different Burgers vectors are similar to their in-plain shear stress distributions. Details of the birefringence contrasts of TEDs depending on the direction of the Burgers vector will be published elsewhere. This result experimentally confirmed that the birefringence image of an off-axis SiC crystal shows a simple correspondence to the in-plane shear stress field in the crystal.

Previously, some researchers have reported that birefringence image contrasts in off-axis SiC wafers correspond to defects such as micropipes and dislocations (Ma & Sudarshan, 2004 ▸; Kato *et al.*, 2017 ▸; Hattori *et al.*, 2018 ▸; Kawata *et al.*, 2021 ▸); the present study reveals that these contrasts represent the in-plane shear stress field of the defects. In addition, reported birefringence image contrasts that are a combination of bright and dark may not always originate from the long-range stress field, as formulated by Tanner & Fathers (1974 ▸), but may derive from the unintentionally induced misorientation of the incident direction from the principal axis and deviation from the crossed-Nicols condition. Furthermore, by comparing the calculated in-plane shear stress and birefringence image observation, one can recognize the detailed characteristics of the dislocations such as the Burgers vector and the line vector.

## 5. Summary

We have theoretically and experimentally demonstrated that the birefringence image contrast variation with the incident direction slightly inclined from the principal axis under conditions slightly deviating from crossed Nicols corresponds to the in-plane shear stress. The current results indicate that the characterization of dislocations in an off-angle wafer such as 4H-SiC for power devices is possible by birefringence imaging.
